# Blood Biomarkers in Ischemic Stroke Diagnostics and Treatment—Future Perspectives

**DOI:** 10.3390/medicina61030514

**Published:** 2025-03-17

**Authors:** Anja Babić, David Bonifačić, Vita Komen, Slavica Kovačić, Melani Mamić, Vladimira Vuletić

**Affiliations:** 1 Department of Neurology, Clinical Hospital Centre Rijeka, 51000 Rijeka, Croatia; 2 Department of Neurology, Faculty of Medicine, University of Rijeka, 51000 Rijeka, Croatia; 3 Department of Diagnostic and Interventional Radiology, Clinical Hospital Centre Rijeka, 51000 Rijeka, Croatia; 4 Department of Radiology, Faculty of Medicine, University of Rijeka, 51000 Rijeka, Croatia

**Keywords:** acute stroke, biomarkers, intravenous thrombolysis, mechanical thrombectomy

## Abstract

Stroke is a leading cause of disability and the second most common cause of death worldwide, with its incidence increasing due to an aging population. Early diagnosis is crucial for timely medical intervention. Biomarkers serve as objective indicators to predict outcomes, monitor treatment responses, and assess prognosis. This review examines the evolving landscape of stroke biomarkers, highlighting their potential clinical applications and the challenges hindering their widespread use. Blood biomarkers are readily accessible and provide insight into the pathophysiological processes underlying stroke. This review focuses on neuronal and glial biomarkers, as well as those associated with inflammation, thrombosis, excitotoxicity, and neuroprotection. Also, it focuses on genetic biomarkers. The timing of biomarker measurement is particularly critical in the early stages of stroke, when rapid decision-making is essential, and it requires systematic investigation. Although numerous molecules have been proposed as stroke biomarkers in recent years, none have yet been integrated into routine clinical practice. Stroke biomarkers hold great promise for enhancing diagnosis, risk stratification, and personalized treatment strategies. However, well-designed studies and rigorous validation are necessary to bridge the gap between research findings and clinical implementation. Integrating biomarkers with existing diagnostic tools could revolutionize stroke management and improve patient outcomes. Continued research into blood biomarkers and their clinical utility remains imperative for advancing stroke care.

## 1. Introduction

Biomarkers can be defined as indicators that objectively assess normal and pathological processes, evaluate the therapeutic response, and predict outcomes [1]. Biomarkers can be molecules detected in body fluids, like cerebrospinal fluid or blood, or physical measurements of tissue. Proteins, metabolites, lipids, and RNA are examples of molecular biomarkers that can be used singly or in combination as panels, scores, or indices. Several biomarkers are already used in everyday clinical decision-making; for example, troponin T helps to set a diagnosis of myocardial infarction, and D-dimer is used for pulmonary embolism, plasma creatinine for kidney function, and C-reactive protein for infections [2].

After a stroke, a series of pathophysiological events occur, progressively causing neuronal damage and ultimately leading to cell death if left untreated [3]. This process follows a time-dependent pattern, with each stage characterized by unique biochemical changes and the release of specific biomarkers that can aid in early diagnosis. The neurovascular unit, as a multicellular structure, represents a potential source of biomarkers for cerebrovascular diseases.

Blood biomarkers have great potential to improve stroke diagnostics, prognosis, and treatment monitoring. They offer a minimally invasive method for early stroke detection and predicting outcomes. This review explores the evolving field of stroke biomarkers, emphasizing their potential clinical applications and the challenges of their implementation. It examines neuronal and glial biomarkers, along with those linked to inflammation, thrombosis, neurotransmitters, and neuroprotection, all of which are protein-based. Additionally, this review addresses genetic biomarkers and highlights the integration of genomic data with proteomics to advance biomarker discovery. Despite previous research, there are still significant difficulties in their clinical application. Therefore, this review discusses future directions in biomarker research. Integrating proteomics and genomics has the potential to improve stroke diagnostics and prognosis. Biomarkers have shown promise as valuable tools that can supplement current diagnostic techniques, offer deeper insights into stroke pathophysiology, and support personalized treatment approaches.

## 2. Acute Ischemic Stroke

The abrupt onset of a focal neurological deficit affecting a particular vascular area of the brain, retina, or spinal cord is what defines an acute stroke. Hemorrhagic and ischemic stroke are the two types of strokes, with ischemic stroke accounting for up to 85% of all strokes [4]. Acute ischemic stroke (AIS) happens when a blood vessel is blocked by a clot, resulting in a restricted blood supply [5].

Hospitals all around the world vary in their diagnostic and treatment capabilities for AIS, based on location and available resources. Recognizing stroke symptoms is a critical first step in ensuring patients receive appropriate treatment. The phrase “time is brain” remains highly relevant, as time plays a vital role in every aspect of AIS.

Brain imaging should be performed as promptly as possible, ideally within 20 min of the patient’s arrival at the hospital [6]. Neuroimaging in the acute stroke setting remains predominantly CT-based. Computed tomography (CT) of the brain is a fast, readily available, and affordable imaging technique [7]. CT protocols, which include a CT scan of the brain, CT angiography (CTA), and CT perfusion (CTP), are used to detect early signs of acute infarction and large vessel occlusion (LVO). Although magnetic resonance imaging (MRI) offers superior sensitivity for detecting AIS compared to other imaging modalities, its limited availability often confines its use to follow-up assessments [8].

Treatment for patients with acute ischemic stroke is guided by the time interval from the onset of stroke to the initiation of treatment. Intravenous thrombolysis (IVT) and endovascular therapy (EVT) are now available treatments for patients diagnosed with AIS who meet specific criteria [9,10,11]. Alteplase is a thrombolytic agent that is manufactured by recombinant DNA technology; it also is referred to as tissue plasminogen activator (rtPA). Alteplase at a dose of 0.9 mg/kg (maximum 90 mg) is administrated as a 10% bolus followed by a 1 h continuous infusion. Alteplase significantly increases the odds of an excellent outcome when given within 4.5 h. Tenecteplase is a genetically modified form of alteplase with an increased resistance to plasminogen activator inhibitor 1, a greater fibrin specificity, and a longer half-life. It is given as a single bolus administration. The Tenecteplase versus Alteplase before Thrombectomy for Ischemic Stroke (EXTENTIA-TNK) trial demonstrated that tenecteplase administration resulted in a higher reperfusion rate and a better functional outcome than alteplase in patients with AIS eligible for EVT [5]. The narrow therapeutic time window and low recanalization rate of intravenous thrombolysis in LVO led to the development of endovascular therapy, which has revolutionized care of AIS [3]. Mechanical thrombectomy entails passing an intra-arterial catheter from a peripheral puncture (usually in the femoral artery) into an intracranial artery and mechanically removing an occluding thrombus, in that way allowing the restoration of the blood flow to the brain [6]. Mechanical thrombectomy is the treatment of choice for patients with an acute onset of stroke symptoms, NIHSS ≥6, due to an imaging-proven LVO in anterior circulation up to 6 h and in posterior circulation (basilar or posterior cerebral artery) up to 24 h after symptom onset. As with rtPA, the benefit of endovascular thrombectomy is highly time-dependent.

Current treatments primarily focus on recanalizing the occluded blood vessels, but they remain inadequate or inaccessible for many stroke patients. Approximately 10–15% of patients are eligible for intravenous thrombolysis and 10% for mechanical thrombectomy [12,13]. Therefore, it is imperative to explore novel therapies for AIS.

## 3. Materials and Methods

This article is a narrative review that comprises literature on blood biomarkers in acute ischemic stroke. A comprehensive search of the literature was conducted on PubMed to locate all pertinent studies published from January 2014 to August 2024. The search strategy was performed using the terms “acute ischemic stroke”, “blood biomarkers in stroke”, “neurovascular unit”, “blood brain barrier”, “astrocytes”, “microglia”, “inflammatory mediators in stroke”, and “genetic biomarkers”. Only full-text original articles published in English were included. Meeting summaries, comments, and secondary analyses were excluded. Data extraction was performed, and the findings were categorized into sections that explored the neurovascular unit (NVU), pathophysiological processes in ischemic stroke, and blood biomarkers in acute ischemic stroke where various biomarkers were described individually.

## 4. Neurovascular Unit

The neurovascular unit concept was first introduced in 2001 by the Stroke Progress Review Group of the National Institute of Neurological Disorders and Stroke [14]. The neurovascular unit is composed of neurons, astrocytes, and microglia, as well as vascular components such as endothelial cells, pericytes, vascular smooth muscle cells, and the basal lamina matrix within the brain’s blood vessels. Part of the NVU is the blood–brain barrier (BBB). The capillary basement membrane, astrocyte end-feet, pericytes, and endothelial cells (ECs) make up the BBB. Together, these elements protect the brain from toxins, filter dangerous chemicals in the blood, and provide essential nutrients to brain tissue [15].

Interactions between the components of the NVU are very important. ECs in the brain are unique because they form tight junctions (TJs) with one another, creating a highly selective barrier. TJs are composed of transmembrane proteins that limit the passive movement of cells and molecules between the blood and the brain, controlling this transfer based on factors such as size, surface charge, and lipid solubility [16]. Endothelial cells (ECs) have a dynamic regulatory mechanism that enables the two-way transport of substances between the blood and the brain [17,18]. Pericytes (PCs) are contractile mural cells that play a critical role in modulating capillary blood flow [18,19]. They regulate the BBB and modulate immune cell trafficking in the brain [20,21]. Additionally, PCs are involved in angiogenesis, which is essential for restoring blood supply. The loss of PCs results in a decrease in the expression of specific TJ proteins, leading to BBB disruption [22]. Vascular smooth muscle cells (VSMCs) play a crucial role in maintaining basal vascular tone, regulating vascular reactivity, and ensuring autoregulation. They surround the arterial walls in a few concentric layers. Astrocytes, the most common glial cell type in the central nervous system, exhibit significant variation in their distribution, function, and structure across different regions of the brain [23]. They have numerous end-feet processes that wrap around blood vessels to form the outermost structure of the BBB [24]. Astrocytes can influence the inflammatory response by releasing pro- and anti-inflammatory cytokines, and chemokines, thereby interacting with microglia [23]. Astrocytes are essential for regulating the transport of nutrients and neurotransmitters after they pass through the BBB. Microglia are immune cells in the CNS that clear debris and promote repair [25]. Also, these cells interact with endothelial cells to assist in regulating the BBB. Neurons release different signaling molecules and neurotransmitters to communicate with other components of the NVU to fulfill the metabolic needs of the brain. The basal lamina matrix plays a crucial role in maintaining vascular integrity. It is comprised of the protein laminin, collagen IV, and heparan sulfate proteoglycans [26].

## 5. The Pathophysiological Processes of the NVU in Ischemic Stroke

Cells of the NVU work together to preserve the integrity of the CNS. Following ischemic stroke, interactions between components of the NVU are disrupted. After a stroke, the initial damage, known as the ischemic core, results from the deprivation of oxygen, glucose, and other essential nutrients, leading to neuronal cell death. If treatment is delayed or not administered, this primary injury spreads to surrounding brain regions, causing secondary injury and forming the ischemic penumbra. The secondary injury arises from a cascade of events, including oxidative stress, inflammation, excitotoxicity, calcium imbalance, endoplasmic reticulum stress, lactate accumulation, mitochondrial dysfunction, and electrolyte disturbances, ultimately leading to further neuronal cell death [27]. The activation of endothelial cells and pericytes leads to BBB disruption, a loss of tight junctions, and increased permeability and therefore cerebral edema [28]. The primary factors contributing to BBB dysfunction include neutrophil infiltration, oxidative stress, decreased tight junction protein levels, and microvascular cell death [27]. This disruption also allows blood proteins and peripheral immune cells to contribute to further injury of brain parenchyma. Therefore, those immune cells release cytokines and proteases. Microglia become activated in response to ischemic injury, releasing pro-inflammatory cytokines such as IL-6, IL-1β, and TNF-α [29]. In response to ischemia, astrocytes become reactive, releasing various cytokines, chemokines, and neurotrophic factors, while upregulating the expression of intermediate filament proteins like glial fibrillary acidic protein (GFAP) [30]. Aquaporin-4 (AQP4) water channels are crucial for maintaining brain water homeostasis and are located on the end-feet of astrocytes. After ischemia, pro-inflammatory mediators contribute to an alteration in AQP4 expression, additionally causing cerebral edema [31]. Astrocytes also interact with microglia to modulate the inflammatory response. Astrocytes express transporters for neurotransmitters like glutamate and GABA, enabling them to clear these molecules from the synaptic cleft and prevent neuronal overstimulation. This regulation is essential for maintaining synaptic plasticity. During ischemia, neurons and astrocytes release excessive glutamate into the extracellular space [32]. Astrocytes clear glutamate from synapses to avoid excessive receptor activation. However, ischemic injury disrupts astrocytic glutamate transporters, causing glutamate to accumulate around neurons, which leads to their damage [33].

## 6. Blood Biomarkers in Acute Ischemic Stroke

Biomarkers are objective indicators that are used to predict results, evaluate treatment responses, and determine whether processes are normal or pathological [3]. For a biomarker to have clinical value, it must demonstrate high sensitivity and specificity to accurately identify an existing disease, while excluding conditions that are not present [34]. While some blood biomarkers are already used in clinical practice, unlike in cardiovascular diseases no single biomarker is currently available for ischemic stroke. Biomarkers may have a role in stroke diagnostics, treatment, and outcome prediction. Specific blood biomarkers that can predict stroke onset time and identify large vessel occlusion can be very helpful in stroke diagnostics and the identification of patients eligible for reperfusion therapies. In addition, stroke biomarkers should be able to differentiate between intracerebral hemorrhage and acute ischemic stroke. The number of blood biomarkers in acute stroke is constantly increasing, but there is no specific biomarker that can be used in decision-making in clinical practice as yet.

This review focuses on those blood biomarkers with the potential to influence clinical practice in acute ischemic stroke (Table 1). They are divided into six groups: neuronal and glial biomarkers, inflammatory biomarkers, thrombosis biomarkers, excitotoxicity and neurotransmitter biomarkers, and neuroprotective and genetic biomarkers.

### 6.1. Neuronal and Glial Biomarkers

**Figure 1:**
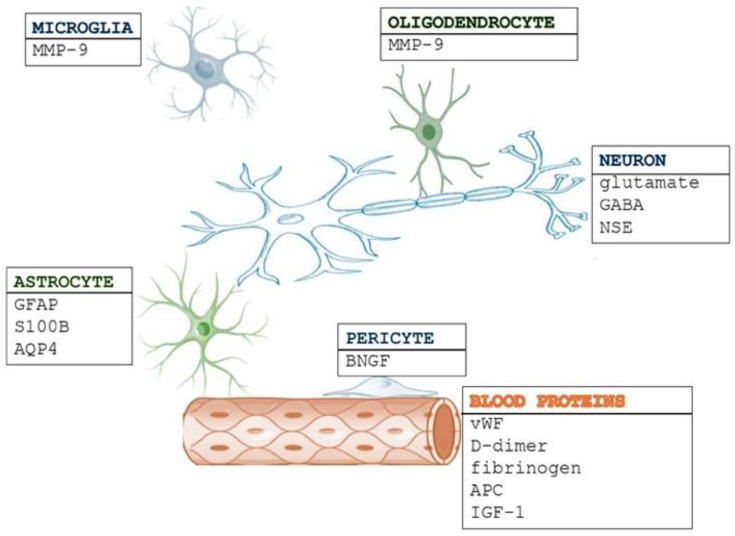
Biomarkers in stroke. Biomarkers have a crucial role in stroke diagnostics, prognosis, and treatment monitoring. Neuronal and glial biomarkers reflect injury to neurons and glial cells. Blood proteins circulate in the bloodstream and reflect systemic responses to stroke, including inflammation, coagulation, oxidative stress, and endothelial dysfunction. These biomarkers provide valuable insights into the underlying processes of stroke and potential related complications.

Neuronal and glial biomarkers are essential in stroke pathophysiology (Figure 1). Neuronal biomarkers indicate direct neuronal damage, axonal injury, and synaptic dysfunction, while glial biomarkers signify blood–brain barrier (BBB) disruption, neuroinflammation, and the activation of astrocytes or microglia. The interaction between these biomarkers provides valuable insights for diagnosis, prognosis, and potential therapeutic interventions.

Glial fibrillary acidic protein (GFAP) is an intermediate filament protein primarily found in astrocytes [50]. Under normal physiological conditions, it is not released into the bloodstream. In healthy individuals, serum GFAP levels remain undetectable unless necrosis and cell breakdown occur, as seen in cases of ischemic stroke or intracranial hemorrhage [51]. Recent research has extensively explored the potential of GFAP as a biomarker in acute stroke. Multicenter studies have demonstrated that GFAP is released more gradually in ischemic stroke compared to its rapid increase in hemorrhagic stroke [35,52,53,54]. In ischemic stroke patients, serum GFAP levels begin rising within 8 h after stroke onset, peaking between days 2 and 5. Conversely, in hemorrhagic stroke, GFAP can be detected in the blood within a few hours due to the rapid breakdown of the blood–brain barrier and early neuronal damage. The slower release of GFAP in ischemic stroke is attributed to the initial preservation of brain cell structure and the blood–brain barrier, with cell necrosis and lysis typically occurring 6–12 h after vessel blockage, leading to a delayed peak in GFAP levels around 48–72 h post-stroke onset [55]. GFAP detection in blood samples is commonly performed using enzyme-linked immunosorbent assays (ELISAs) [38]. A point-of-care GFAP testing platform in ambulances could aid in the rapid triage of stroke patients. A blood test for GFAP detection may enable the early identification of stroke type, allowing timely and appropriate treatment before hospital arrival. Several medications influence GFAP levels by modulating astrocyte activation and neuroinflammation. Corticosteroids suppress astrocyte reactivity, lowering GFAP levels in neuroinflammatory conditions [56]. Statins exhibit neuroprotective properties, reducing GFAP expression in stroke and neurodegenerative diseases [57]. Additionally, drugs such as levodopa and memantine may affect GFAP levels in Parkinson’s and Alzheimer’s disease, respectively [58,59]. While GFAP shows promise as a diagnostic tool for optimizing stroke management in acute settings, further research is necessary to address unresolved variations in findings. Key challenges include establishing age- and gender-specific reference ranges in healthy individuals, defining clinically relevant cutoff values, and standardizing measurement techniques. Moreover, large-scale multicenter studies are essential to assess GFAP’s clinical utility across various conditions. Overcoming these challenges will be crucial for integrating GFAP testing into routine clinical practice.

S100 calcium-binding protein B (S100B) is an intracellular protein primarily found in astrocytes within the nervous system, where it plays a crucial role in calcium regulation [36]. It has been extensively studied in stroke patients over the years. Under normal conditions, S100B is present in the blood at low concentrations. However, it is released in response to neuronal damage, astrocyte activation, and blood–brain barrier disruption. A cross-sectional observational study found that serum S100B levels were significantly elevated in ischemic stroke patients, with a strong correlation between biomarker levels and infarct size [60]. Venous blood samples were collected from all patients within 24 h of hospital admission, and S100B concentrations were measured using standard ELISA kits. Higher S100B levels have been associated with poorer outcomes in stroke patients. Additionally, a prospective two-center study revealed that increased S100B serum concentrations following mechanical thrombectomy correlate with the extent of ischemic tissue damage [61]. S100B can also serve as a marker for detecting early complications in acute stroke, such as hemorrhagic transformation after intravenous thrombolysis [62]. As an indicator of parenchymal brain damage, S100B is valuable in predicting stroke outcomes and has been shown to correlate with infarct size [63].

A water channel protein called aquaporin-4 (AQP4) is present in the end-feet of astrocytes that border ventricular walls and capillary vessels. AQP4 channels play a key role in maintaining water homeostasis by regulating the transport of water. It has an important role in regulating the water balance in the central nervous system that depends on the type of brain edema. In ischemic stroke, the presence of edema is a key prognostic factor, significantly influencing morbidity and mortality [64]. Aquaporin-4 (AQP4) can be detected in the blood after a stroke using ELISA or other immunoassay techniques, such as Western blotting and mass spectrometry. Research indicates that AQP4 levels in the blood may peak within 24 to 48 h following a stroke, coinciding with the progression of BBB disruption and astrocyte damage. AQP4 contributes to astrocytic swelling in cytotoxic edema and aids in the clearance of extracellular water in vasogenic edema [65]. The treatment of ischemic cerebral edema focuses on strategies to alter the fluid content within various brain compartments. Current treatments are primarily limited to osmotic agents such as mannitol and surgical decompression, but these approaches do not address the underlying molecular mechanisms. The identification of AQP4 modulators and inhibitors offers promising new therapeutic possibilities, with the potential to more effectively reduce stroke-related morbidity and mortality. Also, AQP4 can be detected in circulation following an ischemic stroke. Lower circulating levels of AQP4 are linked to early neurological improvement in patients. Additionally, baseline AQP4 levels show an inverse correlation with the progression of infarct size [37]. Further research is needed to establish standardized AQP4 detection methods and define clinically relevant cutoff values for stroke diagnosis and prognosis.

Neuron-specific enolase (NSE) is the neuronal isoform of enolase and is typically present in the blood at very low levels under normal conditions. However, NSE levels rise significantly following acute CNS events such as cerebral infarction, subarachnoid hemorrhage, traumatic brain injury, hypoxia, seizures, and cardiac arrest. These conditions involve BBB disruption and neuronal cell damage, causing NSE to leak into the bloodstream [38]. Elevated NSE levels have been linked to larger infarct sizes, poorer neurological outcomes, and higher mortality rates. In ischemic stroke, NSE levels usually peak between 24 and 72 h after onset, correlating with stroke severity and functional outcomes. In hemorrhagic stroke, NSE elevation may be more pronounced due to more extensive neuronal damage. NSE is not specific to stroke and can increase in other CNS disorders, and its levels rise more slowly compared to biomarkers like GFAP or S100B, making it less useful for early stroke diagnosis. The degree of NSE elevation depends on the integrity of the BBB, which varies across stroke subtypes and individual patients. A study on patients undergoing mechanical thrombectomy for acute ischemic stroke found that elevated NSE levels were independently associated with poorer outcomes and symptomatic intracranial hemorrhage at three months, highlighting its potential to predict post-procedural complications [66]. Despite its promise, NSE’s clinical utility is limited by its lack of specificity, as its levels can also rise in other neurological conditions. Future research should focus on combining NSE with other biomarkers (e.g., GFAP, S100B, or inflammatory markers) to improve stroke diagnosis and prognosis, as well as establishing standardized NSE cutoff values for clinical applications.

### 6.2. Inflammatory Biomarkers

Inflammatory processes and oxidative stress play a role in stroke, exerting both protective and harmful effects on brain tissue. Numerous pro-inflammatory and anti-inflammatory pathways are activated during stroke and represent potential therapeutic strategies. Elevated levels of inflammatory cytokines (IL-18, CRP, IL-12, IL-6) in stroke patients are associated with a poor outcome. Anti-inflammatory cytokines can inhibit pro-inflammatory cytokines. Interleukin-10 (IL-10) is an anti-inflammatory cytokine that is produced in response to brain injury. ELISA is the most used method for detecting IL-10 in serum or plasma. IL-10 levels can rise within the first few hours after stroke, although the increase may not be as immediate as other pro-inflammatory cytokines like IL-6 or TNF-α. The peak in IL-10 elevation often occurs within the first 24 to 48 h after stroke onset, coinciding with the onset of neuroinflammation and the immune system’s response to injury. IL-10 is typically elevated in the acute phase as part of the body’s attempt to control the inflammatory response. Active inflammatory cascades can cause further secondary brain damage. As IL-10 helps resolve active inflammatory cascades, it can reduce further damage to brain tissue [39]. Furthermore, almost all hematopoietic cells that invade the brain following injury are subject to the strong and varied effects of IL-10. It helps limit inflammation by decreasing cytokine receptor expression and inhibiting receptor activation [67]. Lower levels of IL-10 are linked to poor functional outcomes in ischemic stroke patients, indicating that IL-10 is a significant prognostic factor [68]. Elevated IL-10 may indicate a protective, anti-inflammatory response, which is important for stroke management and prognosis.

Most inflammatory responses to acute cerebral ischemia are mediated by cytokines, which rise in patients who have had an acute ischemic stroke in the central nervous system (CNS) and in the systemic circulation. Within the CNS, interleukin-6 (IL-6) is primarily produced and/or expressed by glial cells, especially astrocytes and microglia, as well as by neuronal cells [69]. Endothelial cells are another important source of IL-6 in the CNS. Since IL-6 levels rise sharply in stroke patients soon after the ischemic event, it is a crucial inflammatory factor. It is essential as a messenger molecule between the resident cells of the brain parenchyma, leukocytes, and the vascular endothelium [70]. It contributes to neuroinflammation, which can lead to neuronal damage and worsening outcomes. Monitoring IL-6 levels can help track the intensity of this response. Elevated levels of IL-6 in both the CSF and blood are linked to early neurological deterioration and worse outcomes following a stroke [40]. ELISA is the most common and reliable method for measuring IL-6 levels in blood samples. IL-6 levels rise relatively quickly after a stroke due to its role in the acute inflammatory response. It can be detected within the first few hours, usually within 6 h, following stroke onset, and its concentration often correlates with the severity of the injury. As a potential biomarker and therapeutic target, IL-6 offers promising but complex insights into stroke treatment, requiring careful timing and understanding of its dual nature. Tracking IL-6 levels may help in evaluating the effectiveness of anti-inflammatory treatments or interventions. Future studies are crucial to fully elucidate its role and optimize IL-6-based interventions.

### 6.3. Thrombosis Biomarkers

Thrombosis is a key factor in the development of ischemic stroke, as the formation of blood clots blocks cerebral arteries. Identifying biomarkers associated with thrombosis is crucial for assessing risk, enabling early diagnosis, predicting outcomes, and monitoring treatment in stroke patients. These biomarkers provide insights into platelet activation, coagulation processes, fibrinolysis, and endothelial dysfunction, all of which play a role in the progression of stroke.

Fibrinogen is a soluble plasma glycoprotein produced by the liver that acts as a blood coagulation factor. It circulates in the plasma at high concentrations (2–4 mg/mL) and remains soluble until coagulation occurs, when thrombin catalyzes its conversion into fibrin, forming the matrix of a blood clot [71]. Fibrinogen also plays a role in platelet aggregation [72]. Elevated plasma fibrinogen levels are associated with thrombotic activity and atherosclerosis, affecting arteries like those in the coronary, carotid, and peripheral regions [41]. However, fibrinogen is not specific to stroke, as increased levels are also seen in inflammatory conditions, infections, and cardiovascular diseases. Numerous studies have shown that elevated fibrinogen during the acute phase of ischemic stroke (IS) correlates with poor clinical outcomes. For example, higher fibrinogen levels at hospital admission are linked to worse functional recovery [73,74,75]. Research on 153 patients revealed that those with acute IS had significantly higher fibrinogen levels (>4 g/L) than those with stroke risk factors but no prior stroke history. Fibrinogen levels were measured coagulometrically using an automated analyzer. Fibrinogen levels may start increasing within the first few hours following a stroke. Notably, patients with atherosclerotic strokes or strokes of unknown cause had higher fibrinogen levels compared to other stroke types [76]. Fibrinogen may serve as a prognostic marker for both stroke recovery and the risk of recurrent strokes. While studies have linked high fibrinogen to unfavorable outcomes, its diagnostic reliability is debated. Future research should explore combining fibrinogen with other biomarkers for a more accurate stroke diagnosis and risk assessment.

D-dimer, a product of plasmin-mediated fibrin degradation, is typically present at low concentrations in healthy individuals but rises significantly during acute thrombotic events [42]. Despite its advantages, D-dimer’s clinical use is limited by its lack of specificity. Elevated levels can be influenced by various factors like inflammation, infections, cancer, and venous thromboembolism, making interpretation challenging. This can sometimes lead to false-positive results in stroke diagnosis. A systematic review of 19 studies found that elevated D-dimer levels were linked to worse functional outcomes and higher mortality [77]. Additionally, a cohort study in the acute phase of IS showed that higher D-dimer levels at admission and 24 h later were associated with larger infarct sizes and poorer short-term outcomes [42]. Plasma D-dimer levels were measured using particle-enhanced immunoturbidimetric assays. These findings suggest that D-dimer may be useful for stroke risk assessment, prognosis, and severity, but further research is needed to refine its clinical applications. Given its lack of specificity, combining D-dimer with other biomarkers could enhance stroke diagnosis, and establishing subtype-specific D-dimer cutoffs could help differentiate stroke from other thrombotic or inflammatory conditions.

Von Willebrand factor (vWF), primarily produced by endothelial cells, plays a crucial role in platelet adhesion and aggregation, as well as thrombus formation. It is mostly produced by endothelial cells. It is essential in platelet accumulation and activation in stenotic arteries, contributing to acute thrombotic occlusion [78]. Increased vWF levels are indicative of endothelial injury or dysfunction. A widely used method in clinical laboratories for vWF detection is the immunoturbidimetric assay. High vWF levels may be linked to increased stroke severity and poor clinical outcomes. Elevated vWF levels detected immediately after lysis and at 24 h post-therapy have been identified as independent prognostic factors for poor functional outcomes at 90 days in ischemic stroke patients [43]. Although vWF is a valuable biomarker for evaluating endothelial damage and thrombosis in stroke, it lacks specificity because it can also be elevated in other conditions, such as cardiovascular diseases, inflammation, and other thrombotic disorders. It should be combined with other biomarkers to improve the accuracy of stroke diagnosis and prognosis. Higher vWF levels are linked to poorer outcomes and can assist in predicting stroke severity and recovery. However, due to its lack of specificity, it is important to use vWF alongside other diagnostic methods.

### 6.4. Excitotoxicity and Neurotransmitter Biomarkers

Glutamate is the main excitatory neurotransmitter in the CNS. During a stroke, there is an increased release of glutamate coupled with impaired reuptake, leading to a rapid rise in glutamate levels in the ischemic regions of the brain [79]. Neuronal damage brought on by an excess of glutamate being released and excitatory plasma membrane receptors being activated is known as excitotoxicity [80]. During a stroke, glutamate concentrations in the brain can rise to 10 times higher than normal, creating a significantly greater glutamate concentration in the brain compared to the blood [44]. ELISA can be used to detect glutamate through antibodies specifically designed to bind to glutamate molecules. Glutamate levels in the blood rise rapidly after a stroke, typically within minutes to hours following onset. Plasma glutamate levels in humans have been shown to correlate with the ischemic lesion size on CT or MRI scans and neurological outcomes within the first 24 h after a stroke [81]. Additionally, elevated blood glutamate levels have been closely associated with the development of acute lung injury, a severe complication of stroke and a marker of poor prognosis [82]. The blood glutamate level can serve as a marker for stroke severity. Measuring blood glutamate levels could potentially guide early therapeutic interventions, particularly in high-risk patients, to mitigate the downstream effects of excitotoxicity and improve outcomes.

Gamma-aminobutyric acid (GABA) is the main inhibitory neurotransmitter in the CNS that works to decrease glutamate release brought on by ischemia and depolarization [45]. Following ischemic stroke, an imbalance occurs between excitatory and inhibitory neurotransmission. GABA can be detected in blood by the immunoassay-based method or high-performance liquid chromatography (HPLC). In the acute phase of ischemic stroke, neurons and surrounding cells attempt to compensate for increased excitatory signals by enhancing the release of GABA. GABA’s function opposes that of glutamate; it can counteract glutamatergic neurotoxicity during ischemia [83]. This compensatory mechanism aims to protect neurons by reducing excitatory signaling. Due to its role in reducing neuronal hyperactivity, GABA presents a potential therapeutic target for neuroprotection [45]. The correlation between GABA concentrations in blood plasma and cerebrospinal fluid is weak. A decrease in blood plasma GABA levels may indicate a poorer prognosis for patients with progressive stroke. The potential of GABA as both a biomarker and a therapeutic target remains an active focus of research.

### 6.5. Neuroprotective Biomarkers

While recanalization therapy helps in reopening blocked blood vessels, neuroprotective agents play a crucial role in preserving the function of neurons surrounding the damaged brain tissue, thereby reducing post-stroke deficits. Interest in understanding and developing neuroprotective agents is steadily increasing.

Activated protein C (APC) is an anti-inflammatory and anticoagulant factor known for its neuroprotective role in ischemic stroke. It is generated through the activation of protein C, a glycoprotein primarily produced in the liver and a key component of the natural anticoagulant system. The activation of protein C occurs when the thrombin–thrombomodulin complex interacts with the surface of vascular endothelial cells, converting it into APC. Once activated, APC functions alongside free protein S, phospholipids, and calcium to inactivate factor Va and factor VIIIa at specific arginine cleavage sites [84]. This process reduces prothrombin activation, subsequently decreasing thrombin production and exerting an anticoagulant effect. ELISA is a highly sensitive method that detects APC in blood, but due to rapid clearance from the circulation, its detection requires careful sample handling. APC levels gradually increase in the first 24 h after stroke onset as the body attempts to restore its anticoagulant balance. Besides its anticoagulant role, APC provides neuroprotection by inhibiting cell apoptosis through interactions with endothelial protein C receptor and protease-activated receptor-1 (PAR-1). APC also helps maintain the integrity of the BBB by preventing its breakdown and limiting inflammatory cell infiltration into the brain [46]. Lower circulating levels of insulin-like growth factor-1 (IGF-1) have been linked to a higher risk of stroke and poorer recovery outcomes, further emphasizing the importance of protective factors like APC in stroke prognosis.

The liver produces the majority of IGF-1, which is then transported to various tissues, functioning as an endocrine hormone. IGF-1 is a bioactive peptide with a structure closely resembling insulin, and it plays a vital role in multiple metabolic processes. Additionally, it is essential for nutrition, growth, angiogenesis, and anti-apoptotic mechanisms, contributing to neuroprotection by regulating neuroinflammation and reducing excitotoxicity [47]. ELISA is the method that is frequently used in IGF-1 blood detection. Lower IGF-1 levels post-stroke are associated with worse outcomes, increased inflammation, and a higher mortality risk. Higher IGF-1 levels in recovery correlate with better neuroprotection, cognitive function, and functional recovery. IGF-1 is being explored as a potential biomarker and therapeutic target for stroke rehabilitation.

### 6.6. Genetic Biomarkers

Over time, a variety of substances have been studied as potential biomarkers for stroke diagnosis. Initially, research focused on individual proteins due to their known roles in stroke pathophysiology. However, with the sequencing of the human genome in 2003, this enabled the simultaneous analysis of multiple molecules rather than just single markers. Significant advances in detection methods include mass spectrometry (MS), microarray technology, and polymerase chain reaction (PCR). There is limited research on the genetic factors influencing stroke recovery. The STRONG (Stroke, Stress, Rehabilitation, and Genetics) study examines the genetic factors affecting recovery, particularly cognitive function, depression, and post-traumatic stress disorder (PTSD) one year after stroke [85]. This study provides valuable insights into how genetic factors and stress exposure impact recovery, supporting a holistic approach to stroke rehabilitation that includes genetic screening and stress management to optimize outcomes. Some key genetic markers associated with stroke are brain-derived neurotrophic factor (BDNF), apolipoprotein E (ApoE, variant ε4), angiotensin-converting enzyme (ACE), and nitric oxide synthase 3 (NOS3) [48]. Additionally, the role of microRNAs in repairing neurons in neurological diseases is a promising area of research that may lead to important advancements [49]. Understanding these genetic associations may also identify potential therapy targets to promote neural repair and functional recovery. Developing a basic genetic test could help healthcare providers personalize treatments for patients.

## 7. Discussion

As the population ages, the incidence of stroke is increasing. Early diagnosis is essential for urgent medical treatment. Imaging methods have the main role in acute stroke diagnostics, differentiating ischemic and hemorrhagic stroke, and decision-making regarding acute treatment. In some places, these imaging methods are not available. Blood biomarkers could be used as potential substitutes; they are easily available and can reflect underlying pathophysiological processes during stroke. Biomarkers have become a topic of interest in many studies. The application of biomarkers in the diagnostics and treatment of various diseases is already well known. Despite the identification of several potential blood biomarkers for ischemic stroke, none have yet been adopted in clinical practice. However, with comprehensive research and rigorous validation, blood biomarkers could be developed to improve the management of ischemic stroke patients. Biomarkers of inflammation, endothelial dysfunction, neuronal injury, and oxidative stress have been investigated in stroke patients. Biomarkers used for the early diagnosis of stroke will probably have the most important role in clinical practice. For them to be viable, their levels must increase early after a stroke, with a high specificity for stroke. They should provide additional information which can help clinicians in decision-making in acute stroke. The integration of biomarkers in clinical practice could be crucial for future stroke management. But, despite the advantages of biomarkers, there are also several limitations.

Investigating biomarkers in stroke is challenging due to several factors. Stroke includes multiple types, primarily ischemic and hemorrhagic, each with distinct mechanisms and subtypes, making it difficult to find universal biomarkers. Also, stroke involves multiple cell types (astrocytes, endothelial cells, neurons, pericytes), each contributing differently to the disease pathophysiology. Biomarkers must reflect the interactions within the NVU and distinguish which components are affected, adding complexity to their identification. The BBB regulates biomarkers release into the bloodstream. These biomarkers can be detected in the blood only when the BBB is disrupted. Biomarker levels fluctuate over time, as different molecules are released at various stages of injury, complicating the timing of sample collection. Many potential biomarkers lack specificity for stroke and may be elevated in other conditions. Moreover, many stroke patients have comorbidities that can also affect biomarker levels. The future of genetics in stroke biomarkers offers exciting possibilities for more personalized and accurate methods of diagnosing, predicting, and treating stroke. As we gain a deeper understanding of the genetic factors that affect stroke risk, recovery, and outcomes, genetic biomarkers could play a key role in identifying individuals at a higher risk or those likely to experience more severe effects. Advances in genomic research may uncover new genetic variants linked to stroke, enabling earlier detection and more targeted treatments.

Effective stroke biomarkers must provide rapid results that integrate seamlessly into a clinical setting, where speed is essential for treatment. Further research into and profiling of biomarkers is needed to demonstrate novel biomarkers and their clinical utility.

## 8. Conclusions

Yet there is no specific blood biomarker that can help us in decision-making in patients with acute ischemic stroke. Various markers can be valuable in an acute setting to assist with diagnosis. The identification of blood biomarkers can help in understanding the etiology and determining the subtype of stroke. Also, markers could help in decision-making in reperfusion therapy in acute settings of stroke, in addition to having a significant role in stroke prognosis and predicting outcomes. Blood biomarkers could have a potential role in differentiating ischemic stroke from intracerebral hemorrhage. Their usage should improve stroke management by offering a faster and personalized approach to stroke diagnostics, treatment, and prognosis. Integrating genetic information with other biomarkers could enhance the accuracy of stroke diagnosis and help predict recovery patterns, potentially guiding personalized treatment plans. Further research is needed to validate the clinical applications of specific biomarkers.

## 9. Future Directions

In recent years, various molecules have been suggested as stroke biomarkers, but there is no biomarker used in everyday clinical practice. A single biomarker may not be able to sufficiently represent the complexity of the underlying stroke pathology. Bearing that in mind, the development of panels combining multiple biomarkers could offer a more comprehensive assessment of stroke. Panels could enable tailored plans for acute treatment, diagnostics, and the secondary prevention of stroke in an individualized patient. Combining singular biomarkers into a panel remains an active field of study. Biomarkers could be the new target for therapy in stroke, because not all patients are eligible for reperfusion therapy. Also, as genomic technologies become more accessible and cost-effective, genetic testing could become a routine part of stroke management, allowing healthcare providers to customize care based on each patient’s unique genetic profile. It is important to emphasize the significance of further investigations of blood biomarkers in stroke and their role in clinical practice.

## Figures and Tables

**Table 1:** Stroke biomarkers.

Category	Biomarker	Clinical Significance	References
Neuronal and glial biomarkers	GFAP	helps differentiate stroke from other brain injuries	Perry L.A. et al., (2019) [35]
	S100B	glial damage marker; associated with BBB disruption	Michetti F. et al., (2023) [36]
	AQP4	regulates brain water balance and edema formation after stroke	Ramiro L. et al., (2020) [37]
	NSE	marker of neuronal damage; correlates with stroke severity	Freitas T.E. et al., (2024) [38]
Inflammatory biomarkers	IL-10	anti-inflammatory; neuroprotective role	Garcia J.M. et al., (2017) [39]
	IL-6	correlates with infarct size and poor prognosis	Aref H.M.A. et al., (2020) [40]
Thrombosis biomarkers	fibrinogen	high levels linked to clot formation	Abebe E.C. et al., (2024) [41]
	D-dimer	elevated in cardioembolic stroke	Abbas N.I. et al., (2021) [42]
	vWF	predicts stroke recurrence	Toth N.K. et al., (2017) [43]
Excitotoxicity and neurotransmitter biomarkers	glutamate	high levels indicate neuronal injury and stroke severity	Kaplan-Arabaci O. et al., (2022) [44]
	GABA	plays a role in neuroprotection and recovery	Michalettos G. et al., (2022) [45]
Neuroprotective biomarkers	APC	reduces inflammation, potential therapeutic target	Griffin J.H. et al., (2018) [46]
	IGF-1	modulating neuroinflammation and mitigating excitotoxicity	Du H. et al., (2023) [47]
Genetic biomarkers	ApoE ε4 gene	associated with younger age at stroke onset	Zhang K. et al., (2023) [48]
	microRNA	regulates neuroinflammation, brain repair, and stroke severity	Xu W. et al., (2018) [49]
